# The age at natural menopause of Chinese Han and Tibetan women

**DOI:** 10.3389/fendo.2025.1584267

**Published:** 2025-08-01

**Authors:** Shiqi Chen, Liangbo Yang, Furui Chen, Yu Qiu, Jing Zhang, Jesse Li Ling, Qiaolan Liu, Yan Gong

**Affiliations:** ^1^ Department of Obstetrics, Sichuan Provincial Women’s and Children’s Hospital, The Affiliated Women’s and Children’s Hospital of Chengdu Medical College, Chengdu, Sichuan, China; ^2^ Department of Reproductive Medicine, Sichuan Jinxin Xinan Women and Children’s Hospital, Chengdu, Sichuan, China; ^3^ Reproductive Medicine Center, Sichuan Provincial Women’s and Children’s Hospital, The Affiliated Women’s and Children’s Hospital of Chengdu Medical College, Chengdu, Sichuan, China; ^4^ School of Clinical Medicine, Chengdu Medical College, Chengdu, Sichuan, China; ^5^ Reproductive Endocrinology, Department of Obstetrics and Gynecology, West China Second University Hospital, Sichuan University, Chengdu, Sichuan, China; ^6^ Center of Medical Genetics, West China Second University Hospital, Sichuan University, Chengdu, Sichuan, China; ^7^ West China School of Public Health and West China Fourth Hospital, Sichuan University, Chengdu, Sichuan, China

**Keywords:** ethnicity, Chinese Han, Chinese Tibetan, postmenopausal, age at natural menopause

## Abstract

**Background:**

Studies have reported that the age at natural menopause may vary between different ethnic groups. Chinese Han and Tibetan women exhibit substantial differences in their living environment, dietary habits, and lifestyle; however, few studies have specifically investigated the potential differences in their age at natural menopause. This study was designed to identify the discrepancy in the age at natural menopause between postmenopausal women of Chinese Han and Tibetan ethnicities.

**Methods:**

A cross-section study has been conducted on 5,562 Han and 1,049 Tibetan postmenopausal women who were recruited by the China Multi-ethnic Cohort study from May 2018 to September 2019. The participants have resided in Chengdu City and Aba Prefecture, respectively, with ethnicity set as the primary exposure variable. Linear and multinomial logistic regression model was used to assess the association of the age at natural menopause with influencing factors and odds ratios for the association between premature ovarian failure, early and late menopause with the ethnicity, and influencing factors.

**Results:**

The mean age at natural menopause of Han women was 0.74 year earlier than Tibetan women (*P* = 0.003). The Han women also showed a lower likelihood for experiencing late menopause with an adjusted odds ratio (95% confidence intervals) of 0.54 (0.34, 0.88) compared with the Tibetan women. Factors associated with later menopause have included older age at survey, ever married, high school and above educational level, annual household income of ≥200,000 RMB, no smoking, habit of eating spicy food, no history of severe food shortage, more gravidity, less parity, use of intrauterine device, and no history of using oral contraceptives.

**Conclusions:**

The age at natural menopause of Chinese Han women was earlier than that of Chinese Tibetan women. Demographic, lifestyle, dietary habits, and reproductive factors may influence the age at natural menopause.

## Introduction

Natural menopause is defined as the permanent cessation of menstruation due to the exhaustion of ovarian reserve, which is diagnosed as amenorrhea lasting for 12 consecutive months (1). Age at natural menopause (ANM) may reflect the reproductive lifespan of the ovary (2) and is 46 to 52 years old as reported worldwide (3). The ANM has been associated with health conditions. Earlier ANM (younger than 45 years old) has been associated with cardiovascular disease, type 2 diabetes, osteoporosis, and early death (4–6), while later ANM may increase the risk of endometrial cancer (7). Factors influencing the ANM have included genetics, ovarian diseases or surgery, environment, and unhealthy lifestyle (3, 8). Research on the ANM and its associated factors may facilitate the prevention of the disease, improving the quality of life and making reproductive plans.

The ANM has appeared to vary across different regions and ethnic groups (3). Studies have found that the ANM is earlier in Africans, Latin Americans, and Asians and is later in Europeans, Australians, and Americans (3, 9), which suggested that race/ethnicity is an independent predictor for the ANM (2, 9, 10), while others have reported that the difference in ANM among different ethnic groups may be explained to a large extent by controlling covariates such as socio-demography, lifestyle, and health conditions (11, 12). Therefore, whether ethnicity is an independent factor for the ANM has remained controversial.

China is a country with 56 ethnic groups. The different living environments, lifestyles, health conditions, and genetic backgrounds may have all contributed to the discrepancy in the ANM between different ethnic groups. Some studies have reported the ANM of Chinese women (13–15). Some have reported the average menopausal age for ethnic Han women to be later than that for Uyghur women from Xinjiang (48.11 vs. 47.01) (16) but was earlier than Tibetan women from Yunnan (48.30 vs. 48.90) (17). It is interesting to note that both natural and pathologically menopausal women have been included in these studies (16, 17). Only one study has reported that the ANM of Han women was earlier than that of Miao women from Guiyang (47.83 vs. 48.05) (18).

Sichuan is a Chinese province where all 56 ethnic groups reside and is also the second largest Tibetan residential area in China. In Chengdu (basin), the capital of Sichuan province, the major inhabitants are ethnic Hans. In Aba Prefecture (plateau), with a high altitude, low oxygen level, and intense ultraviolet radiation, the major inhabitants are Tibetans. Most Tibetans are engaged in animal husbandry, and their diets are mainly characterized by high-caloric and high-protein (19). Based on existing literature and documented ethnic differences (17), this study proposes the research hypothesis that the ANM of Han women was earlier than that of Tibetan women. Using data from the China Multi-ethnic Cohort (CMEC), we investigated the differences in the ANM between the Han and Tibetan postmenopausal women in Sichuan as well as the influencing factors.

## Materials and methods

### Study population

Our study utilized baseline data from the CMEC, a prospective cohort study aimed to investigate the genetic and environmental determinants of non-communicable diseases across diverse ethnic groups in Southwest China. The participants (*n* = 99,556; aged 30–79 years) were recruited voluntarily between May 2018 and September 2019 using multistage stratified cluster sampling through local communities. The main contents of the baseline survey have included demographic and socio-economic status, smoking habit, alcohol intake, diet, disease history, physical activity, and reproductive history. All participants completed a full questionnaire based on laptops through face-to-face interviews with audio recording in the local community health service centers or township health centers. On the same day of the survey, data quality inspectors randomly selected questionnaires and evaluated their data quality by listening to the audio recordings. All participants provided written informed consent and retained the right to withdraw at any time without penalty. The CMEC study design and survey methods has been described previously (20). The CMEC study has been approved by the Sichuan University Medical Ethical Review Board (IRB number: K2016038).

For this study, ethical approval was obtained from the Medical Ethics Committee of Sichuan Provincial Women’s and Children’s Hospital (no. 20240903) and then registered in the Chinese Clinical Trial Registry (ChiCTR2400090175). In this study, postmenopausal Han women who lived in Chengdu and postmenopausal Tibetan women who lived in Aba were enrolled from CMEC. Menopause was diagnosed as without menstruation for ≥12 months (1). All participants were permanent residents. The ethnic group was determined by their identity cards. Individuals were excluded for any of the following conditions: missing the information of ANM, history of cancer, hysterectomy, and ovariectomy before menopause.

### Study factors

On the baseline questionnaire, postmenopausal women were asked about the age of last menstruation. Self-reported ANM was the primary outcome. Based on the ANM, menopause was divided into four types: premature ovarian failure (POF) (<40 years), early menopause (EM) (40 to 44 years), normal menopause (NM) (45 to 54 years), and late menopause (LM) (≥55 years) (4, 21).

Ethnicity was the primary exposure variable. Basal characteristics with potential effect on the ANM were included, such as sociodemographic factors (age at survey, marriage status, education level, and annual household income), lifestyle (smoking habit, passive smoking, labor history, pesticide exposure history, coal smoke exposure history, and severe food shortage history), dietary habit (alcohol intake, tea drinking, soft drink consumption, spicy foods, and numb foods), and reproductive history [(age at menarche, number of gravidity and parity, age at first birth, duration of breastfeeding per child, utilization of the intrauterine device (IUD), and utilization of an oral contraceptive (OC)].

### Statistical analysis

Analyses were completed using R-4.2.0 (R Foundation for Statistical Computing). Descriptive statistics were presented as median and interquartile range (IQR) for continuous variables and numbers (percentages) for categorical variables. Kruskal–Wallis *H* test for continuous variables and chi-square test for categorical variables were used to compare the differences in baseline characteristics between Chinese Han and Tibetan postmenopausal women. Linear regression models were used to assess the association of ANM with ethnicity and influencing factors. To further estimate the adjusted regression coefficients (*β*) and 95% confidence intervals (CI), we have used the multivariable linear regression models. Multinomial logistic regression was used to estimate the adjusted odds ratios (ORs) and 95% CI for the association among POF, EM, and LM with ethnicity and the influencing factors. Two-sided *P*-values <0.05 were considered statistically significant. Considering that the recall bias among elderly respondents may have influenced the study outcomes, we conducted a sensitivity analysis by excluding females whose age at survey was over 75 years.

## Results

**Figure 1:**
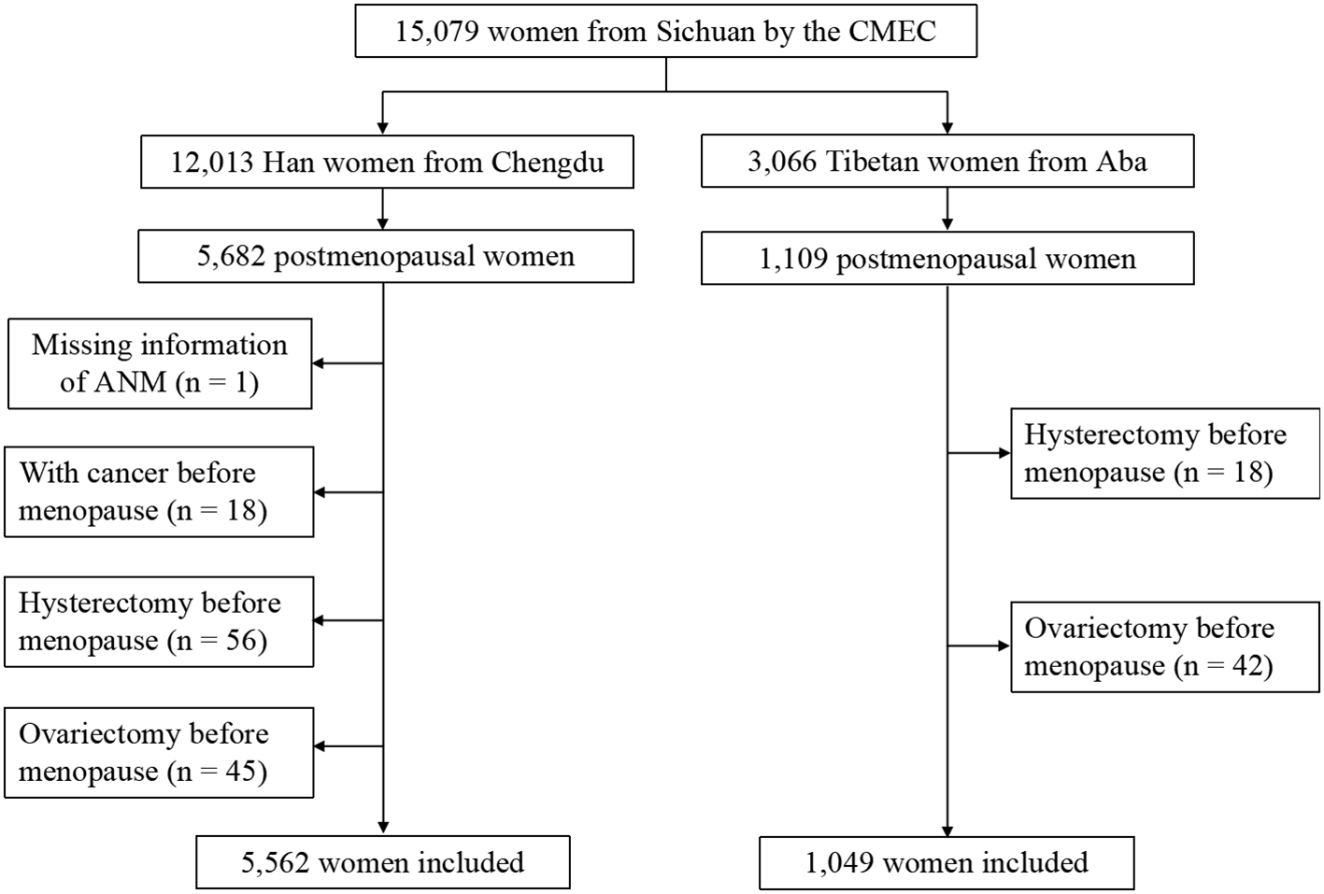
Flowchart of enrollment. Progress of retrospective cohort study including enrollment and exclusion.

A total of 5,562 Han and 1,049 Tibetan postmenopausal women were enrolled, and 180 postmenopausal women were excluded (**Figure 1**). The exposure variables and operational definitions are summarized in **Supplementary Table S1**.

### Menstruation and gestation of the postmenopausal women

**Figure 2:**
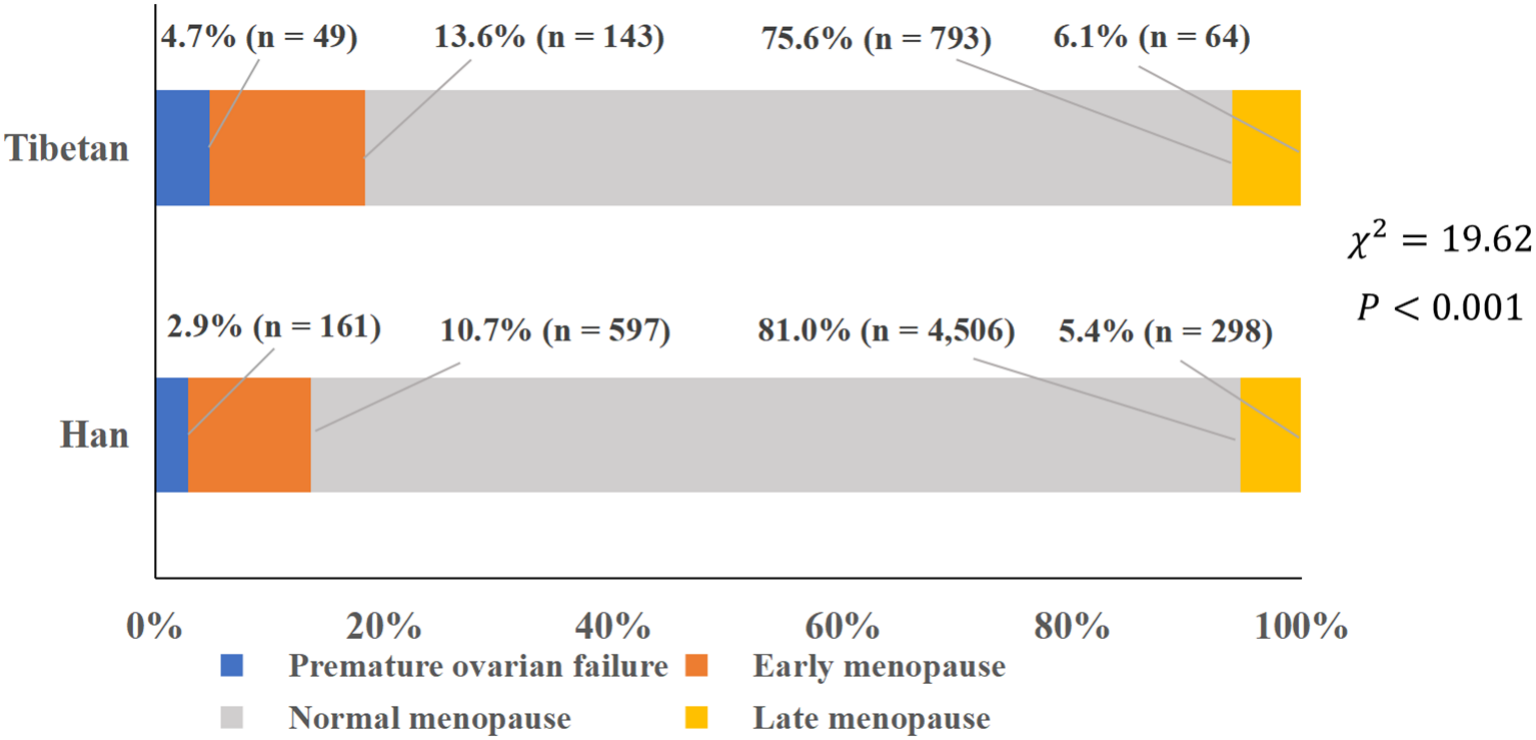
Proportion of various types of menopause. Proportions of premature ovarian failure, early menopause, normal menopause, and late menopause.

In Han postmenopausal women, ANM, the age at first birth, duration of breastfeeding per child, utilization of IUD, and utilization of OC were significantly higher, whereas the age at menarche, number of gravidity, and parity were significantly lower than those of Tibetan postmenopausal women (*P* < 0.001). The proportion of menopausal types was also significantly different between the two groups, with the Han women tending to have more NM and fewer POF, EM, and LM (*P* < 0.001) (**Table 1**, **Figure 2**).

**Table 1:** Menstruation and gestation between Han and Tibetan postmenopausal women.

Variables	Overall (*n* = 6,611)	Han (*n* = 5,562)	Tibetan (*n* = 1,049)	*P-*value
ANM (year)	49.0 (46.0–51.0)	49.0 (46.0–51.0)	49.0 (45.0–50.0)	<0.001
Menopausal type (%)				<0.001
POF	210 (3.2)	161 (2.9)	49 (4.7)	
EM	740 (11.2)	597 (10.7)	143 (13.6)	
NM	5299 (80.1)	4506 (81.0)	793 (75.6)	
LM	362 (5.5)	298 (5.4)	64 (6.1)	
Age at menarche (year)	15.0 (14.0–17.0)	15.0 (14.0–17.0)	15.0 (15.0–17.0)	<0.001
No. of pregnancy	3.0 (2.0–4.0)	3.0 (2.0–4.0)	4.0 (3.0–5.0)	<0.001
No. of parity	1.0 (1.0–3.0)	1.0 (1.0–2.0)	3.0 (2.0–5.0)	<0.001
Age at first birth (year)	23.0 (21.0–25.0)	24.0 (22.0–25.0)	20.0 (19.0–22.0)	<0.001
Duration of breastfeeding per child (month)	6.0 (3.0–12.0)	6.0 (3.3–12.0)	3.4 (1.9–6.0)	<0.001
Utilization of IUD (%)				<0.001
Never	2,447 (37.0)	1,637 (29.4)	810 (77.2)	
Ever	4,164 (63.0)	3,925 (70.6)	239 (22.8)	
Utilization of OC (%)				<0.001
Never	5,913 (89.4)	4,894 (88.0)	1,019 (97.1)	
Ever	698 (10.6)	668 (12.0)	30 (2.9)	

ANM, age at natural menopause; POF, premature ovarian failure; EM, early menopause; NM, normal menopause; LM, late menopause; No., number; IUD, intrauterine device; OC, oral contraceptive.

### Basal characteristics of the Han and Tibetan postmenopausal women

The range of survey age was 37–79 years old for both Han and Tibetan postmenopausal women. In Han postmenopausal women, the age at survey, proportion of ever married, educational level, annual household income, smoking, passive smoking, alcohol intake, spicy foods, numb foods, history of labor, pesticide exposure, coal smoke exposure, and severe food shortage were significantly higher, whereas the proportion of tea drinking and soft drink consumption was significantly lower than those in Tibetan postmenopausal women (*P* < 0.001) (**Table 2**).

**Table 2:** Basal characteristics between Han and Tibetan postmenopausal women.

Variable	Overall (*n* = 6,611)	Han (*n* = 5,562)	Tibetan (*n* = 1,049)	*P-*value
Age at survey (year)	61.0 (54.0–67.0)	61.0 (54.0–67.0)	57.0 (52.0–65.0)	<0.001
Marriage status (%)				<0.001
Never married	32 (0.5)	8 (0.1)	24 (2.3)	
Ever married	6,579 (99.5)	5,554 (99.9)	1,025 (97.7)	
Education level (%)				<0.001
Middle school and below	5,598 (84.7)	4,570 (82.2)	1,028 (98.0)	
High school and above	1,013 (15.3)	992 (17.8)	21 (2.0)	
Annual household income (RMB/year), (%)				<0.001
<20,000	2,244 (34.0)	1,672 (30.1)	572 (54.5)	
20,000–99,999	3,798 (57.5)	3,335 (60.1)	463 (44.2)	
100,000–199,999	476 (7.2)	463 (8.3)	13 (1.2)	
≥200,000	84 (1.3)	83 (1.5)	1 (0.1)	
Smoking (%)				<0.001
No smoking	6,487 (98.1)	5,438 (97.8)	1,049 (100.0)	
Ever smoking	124 (1.9)	124 (2.2)	0 (0.0)	
Passive smoking (%)				<0.001
No	4,042 (61.1)	3,058 (55.0)	984 (93.8)	
Yes	2,569 (38.9)	2,504 (45.0)	65 (6.2)	
Alcohol intake (%)				<0.001
Never drinking	5,101 (77.2)	4,059 (73.0)	1,042 (99.3)	
Ever drinking	1,510 (22.8)	1,503 (27.0)	7 (0.7)	
Tea drinking (%)				<0.001
No	4,557 (68.9)	4,223 (75.9)	334 (31.8)	
Yes	2,054 (31.1)	1,339 (24.1)	715 (68.2)	
Soft drinking (%)				<0.001
No	6,125 (92.6)	5,481 (98.5)	644 (61.4)	
Yes	486 (7.4)	81 (1.5)	405 (38.6)	
Spicy foods (%)				<0.001
No	2,069 (31.3)	1,433 (25.8)	636 (60.6)	
Yes	4,542 (68.7)	4,129 (74.2)	413 (39.4)	
Numb foods (%)				<0.001
No	1,860 (28.1)	1,141 (20.5)	719 (68.5)	
Yes	4,751 (71.9)	4,421 (79.5)	330 (31.5)	
Labor history (%)				<0.001
No	1,877 (28.4)	1,475 (26.5)	402 (38.3)	
Yes	4,734 (71.6)	4,087 (73.5)	647 (61.7)	
Pesticide exposure history (%)				<0.001
No	4,210 (63.7)	3,184 (57.2)	1,026 (97.8)	
Yes	2,401 (36.3)	2,378 (42.8)	23 (2.2)	
Coal smoke exposure history (%)				<0.001
No	4,426 (66.9)	3,489 (62.7)	937 (89.3)	
Yes	2,185 (33.1)	2,073 (37.3)	112 (10.7)	
Severe food shortage history (%)				<0.001
No	4,546 (68.8)	3,517 (63.2)	1,029 (98.1)	
Yes	2,065 (31.2)	2,045 (36.8)	20 (1.9)	

### Linear regression analysis of the ANM

After adjusting the confounders, multiple linear regression analysis showed that the mean ANM of Han postmenopausal women was 0.74 year younger than Tibetan postmenopausal women (*P* = 0.003). Older at survey, ever married, high school and above education level, annual household income of ≥200,000 RMB, no smoking, habit of eating spicy food, never experienced severe food shortage, more gravidity, less parity, utilization of IUD, and never used OC were associated with later ANM (*P* < 0.05) (**Table 3**). The sensitivity analysis restricted to women whose age at survey was ≤75 years (*n* = 6,353) yielded consistent findings with the primary analysis, with no substantial changes in the effect size or significance of the association between ethnicity and the ANM (adjusted *β* = -0.67, 95% CI: -1.17 to -0.16, *P* = 0.009, **Supplementary Table S2**).

**Table 3:** Linear regression analysis of ANM.

Variables	Univariate analysis		Multivariate analysis^a^	
	*β* (95% CI)	*P*-value	*β* (95% CI)	*P*-value
Ethnicity (ref: Tibetan)	0.68 (0.39, 0.97)	<0.001	-0.74 (-1.23, -0.25)	0.003
Age at survey	0.10 (0.09, 0.11)	<0.001	0.14 (0.12, 0.15)	<0.001
Marriage status (ref: never married)	3.23 (1.72, 4.74)	<0.001	2.82 (1.01, 4.64)	0.002
Education level (ref: middle school and below)	0.46 (0.17, 0.75)	0.002	0.60 (0.28, 0.93)	<0.001
Annual household income (ref: <20,000)				
20,000–99,999	0.29 (0.06, 0.52)	0.012	0.20 (-0.04, 0.44)	0.097
100,000–199,999	0.40 (-0.03, 0.82)	0.072	0.18 (-0.26, 0.62)	0.430
≥200,000	1.35 (0.40, 2.30)	0.005	1.09 (0.15, 2.04)	0.024
Smoking (ref: no smoking)	-0.69 (-1.47, 0.08)	0.078	-1.00 (-1.76, -0.24)	0.010
Passive smoking (ref: no)	-0.02 (-0.24, 0.19)	0.842	0.05 (-0.17, 0.28)	0.648
Alcohol intake (ref: never drinking)	-0.13 (-0.38, 0.11)	0.294	-0.20 (-0.46, 0.05)	0.125
Tea drinking (ref: no)	-0.05 (-0.28, 0.17)	0.641	0.20 (-0.04, 0.44)	0.099
Soft drinking (ref: no)	-0.83 (-1.23, -0.43)	<0.001	-0.39 (-0.87, 0.08)	0.103
Spicy foods (ref: no)	0.17 (-0.06, 0.40)	0.140	0.30 (0.04, 0.55)	0.023
Numb foods (ref: no)	0.21 (-0.02, 0.45)	0.074	-0.01 (-0.28, 0.26)	0.956
Labor history (ref: no)	0.41 (0.17, 0.64)	0.001	0.15 (-0.10, 0.40)	0.240
Pesticide exposure history (ref: no)	0.14 (-0.08, 0.35)	0.221	0.09 (-0.16, 0.34)	0.490
Coal smoke exposure history (ref: no)	0.14 (-0.09, 0.36)	0.233	0.01 (-0.23, 0.24)	0.959
Severe food shortage history (ref: no)	0.30 (0.07, 0.52)	0.010	-0.35 (-0.60, -0.11)	0.005
Age at menarche (year)	0.12 (0.07, 0.17)	<0.001	0.05 (0.00, 0.10)	0.073
No. of pregnancy	0.08 (0.03, 0.14)	0.003	0.10 (0.03, 0.18)	0.008
No. of parity	0.02 (-0.04, 0.09)	0.490	-0.33 (-0.45, -0.20)	<0.001
Age at first birth (year)	0.07 (0.03, 0.10)	<0.001	-0.01 (-0.05, 0.03)	0.523
Duration of breastfeeding per child (month)	0.01 (-0.01, 0.02)	0.416	0.01 (-0.01, 0.03)	0.440
Utilization of IUD (ref: never)	0.29 (0.07, 0.51)	0.009	0.52 (0.28, 0.76)	<0.001
Utilization of OC (ref: never)	-0.29 (-0.63, 0.05)	0.100	-0.36 (-0.71, -0.02)	0.038

CI, confidence interval; No., number; IUD, intrauterine device; OC, oral contraceptive.

a Adjusted for ethnicity, age at survey, marriage status, education level, annual income, smoking, passive smoking, alcohol intake, tea drinking, soft drink consumption, spicy foods, numb foods, labor history, pesticide exposure history, coal smoke exposure history, severe food shortages history, age at menarche, number of pregnancy, number of parity, age at first birth, duration of breastfeeding per child, utilization of IUD, and utilization of OC, except for the same variables.

### Multinomial logistic regression of the menopausal types

After adjusting for confounders, older women at survey were less likely to experience POF (OR = 0.92, 95% CI: 0.90–0.94) and EM (OR = 0.95, 95% CI: 0.94–0.96). Additionally, ever married (OR = 0.33, 95% CI: 0.12–0.91), high school and above education level (OR = 0.67, 95% CI: 0.51–0.88), annual household income ≥200,000 RMB (OR = 0.24, 95% CI: 0.06–0.99), habit of eating spicy foods (OR = 0.82, 95% CI: 0.67–0.99), and utilization of IUD (OR = 0.75, 95% CI: 0.62–0.90) were protective factors for EM. By contrast, smoking habit (OR = 1.79, 95% CI: 1.09–2.97), history of severe food shortage (OR = 1.24, 95% CI: 1.02–1.50), more parity (OR = 1.20, 95% CI: 1.09–1.32), and utilization of OC (OR = 1.43, 95% CI: 1.12–1.83) are risk factors for EM. Compared with Tibetan postmenopausal women, Han postmenopausal women were less likely to experience LM (OR = 0.54, 95% CI: 0.34–0.88). Older women at survey were more likely to experience LM (OR = 1.09, 95% CI: 1.07–1.11) (**Table 4**). The sensitivity analysis restricted to women whose age at survey was ≤75 years was consistent with the primary analysis (**Supplementary Table S3**).

**Table 4:** Multinomial logistic regression for non-normal menopausal type.

Variable	POF^a^ (*n* = 210)		EM^a^ (*n* = 740)		LM^a^ (*n* = 362)	
	OR (95% CI)	*P*-value	OR (95% CI)	*P*-value	OR (95% CI)	*P*-value
Ethnicity (ref: Tibetan)	1.31 (0.68, 2.53)	0.424	1.27 (0.88, 1.84)	0.206	0.54 (0.34, 0.88)	0.013
Age at survey	0.92 (0.90, 0.94)	<0.001	0.95 (0.94, 0.96)	<0.001	1.09 (1.07, 1.11)	<0.001
Marriage status (ref: never married)	0.33 (0.07, 1.59)	0.168	0.33 (0.12, 0.91)	0.032	1.10 (0.14, 8.86)	0.926
Education level (Ref: middle school and below)	0.84 (0.53, 1.34)	0.465	0.67 (0.51, 0.88)	0.004	1.16 (0.81, 1.65)	0.411
Annual household income (ref: <20,000 RMB)						
20,000–99,999	0.78 (0.57, 1.07)	0.126	0.95 (0.79, 1.13)	0.544	0.89 (0.69, 1.14)	0.359
100,000–199,999	0.79 (0.42, 1.49)	0.462	1.00 (0.71, 1.42)	0.998	1.11 (0.70, 1.76)	0.643
≥200,000	0.34 (0.05, 2.53)	0.294	0.24 (0.06, 0.99)	0.049	1.71 (0.77, 3.77)	0.184
Smoking (ref: no smoking)	1.50 (0.53, 4.19)	0.443	1.79 (1.09, 2.97)	0.023	0.68 (0.27, 1.72)	0.416
Passive smoking (ref: No)	0.94 (0.68, 1.30)	0.725	1.11 (0.93, 1.32)	0.262	0.86 (0.67, 1.10)	0.221
Alcohol intake (ref: never drinking)	0.75 (0.50, 1.10)	0.141	1.16 (0.95, 1.41)	0.146	1.07 (0.81, 1.41)	0.642
Tea drinking (ref: No)	0.92 (0.65, 1.29)	0.627	0.86 (0.71, 1.04)	0.129	1.05 (0.81, 1.36)	0.696
Soft drinking (ref: No)	1.18 (0.67, 2.07)	0.562	0.94 (0.67, 1.33)	0.727	0.70 (0.41, 1.20)	0.196
Spicy foods (ref: No)	0.80 (0.56, 1.15)	0.232	0.82 (0.67, 0.99)	0.039	0.97 (0.75, 1.26)	0.832
Numb foods (ref: No)	1.20 (0.82, 1.75)	0.344	0.94(0.77, 1.15)	0.552	0.96 (0.72, 1.27)	0.749
Labor history (ref: No)	0.84 (0.60, 1.17)	0.300	0.98 (0.81, 1.18)	0.818	0.83 (0.63, 1.09)	0.177
Pesticide exposure history (ref: no)	0.73 (0.51, 1.06)	0.096	1.03 (0.85, 1.26)	0.733	0.90 (0.68, 1.17)	0.422
Coal smoke exposure history (ref: No)	0.95 (0.68, 1.32)	0.753	0.95 (0.79, 1.13)	0.558	1.08 (0.85, 1.38)	0.538
Severe food shortage history (ref: no)	1.29 (0.89, 1.85)	0.173	1.24 (1.02, 1.50)	0.029	0.89 (0.69, 1.14)	0.343
Age at menarche (year)	1.01 (0.94, 1.08)	0.831	1.00 (0.96, 1.04)	0.894	1.02 (0.97, 1.08)	0.342
Number of pregnancy	1.01 (0.91, 1.12)	0.864	0.96 (0.91, 1.02)	0.228	1.03 (0.96, 1.11)	0.364
Number of parity	1.11 (0.93, 1.31)	0.247	1.20 (1.09, 1.32)	<0.001	0.92 (0.81, 1.04)	0.189
Age at first birth (year)	1.01 (0.95, 1.06)	0.827	1.01 (0.98, 1.04)	0.467	1.03 (0.99, 1.07)	0.173
Duration of breastfeeding per child (month)	1.00 (0.97, 1.03)	0.946	1.00 (0.98, 1.01)	0.881	1.00 (0.98, 1.03)	0.723
Utilization of IUD (ref: never)	0.92 (0.66, 1.29)	0.625	0.75 (0.62, 0.90)	0.002	1.02 (0.80, 1.31)	0.867
Utilization of OC (ref: never)	1.30 (0.83, 2.04)	0.247	1.43 (1.12, 1.83)	0.005	1.25 (0.88, 1.79)	0.214

CI, confidence interval; POF, premature ovarian failure; EM, early menopause; LM, late menopause. OR, odds ratio; No., number; IUD, intrauterine device; OC, oral contraceptive.

a Adjusted for ethnicity, age at survey, marriage status, education level, annual income, smoking, passive smoking, alcohol intake, tea drinking, soft drink consumption, spicy foods, numb foods, labor history, pesticide exposure history, coal smoke exposure history, severe food shortage history, age at menarche, number of pregnancy, number of parity, age at first birth, duration of breastfeeding per child, utilization of IUD, and utilization of OC, except for the same variable.

## Discussion

In this study, an analysis of a large size of samples has suggested that the ANM of Chinese Han postmenopausal women was earlier compared with Tibetan postmenopausal women and they were less likely to suffer LM. Older at survey, ever married, high school and above education level, annual household income ≥200,000 RMB, no smoking, habit of eating spicy foods, never experienced severe food shortage, more gravidity, less parity, utilization of IUD, and never used OC were associated with later ANM.

Whether ANM is a disparity among different ethnic groups is still controversial. Some studies indicated race/ethnicity to be a significant independent predictor for ANM (2, 3, 9, 10). A study involving five racial/ethnic groups has found that, compared with non-Latina Whites, natural menopause have occurred earlier among Latinas and later among Japanese Americans (10). A meta-analysis has indicated that the ANM has varied from 44.6 to 54.5 years across different races, with earlier menopause in women from African, Middle Eastern, some Latin American, and Asian countries and later menopause in women from Europe, Australia, Canada, and USA (3), while others have reported no racial diversity in the ANM after the confounders were controlled, including socioeconomic, lifestyle, and health conditions (11, 12).

We found more gravidity, fewer parity, and never used OC to be associated with later ANM. On the other hand, more parity and use of OC may increase the risk for EM. During gravidity, prevention of ovulation and slow depletion of the ovarian reserve may account for later menopause (22, 23). However, more parity has been associated with earlier ANM and EM in our study, which differed from other reports (10, 21–23). In our study, parity referred to the number of live birth, while for other studies this was the number of children (21) and pregnancies lasting ≥ 6 months (23). Postpartum hemorrhage and Sheehan syndrome are the risks after delivery (24, 25), which then reduced the ovarian reserve. Therefore, we propose this to be the reason for more parity and earlier ANM in our study. The relationship between OC and ANM seems to be controversial. One study has suggested that OC could delay the menopause and prolong the reproductive span (26), while (27) have found that ANM was not affected by OC after adjusting the confounders (28). They have reported that OC was associated with earlier ANM, which was consistent with our study. OC has been used not only for contraception but also in treating menstrual disorders (29). The decreased ovarian reserve in some of these patients might account for their earlier ANM. In keeping with our finding, a Dutch study has found that high-dose OC could advance the onset of menopause (28).

Our study has also found that lower educational level and income may also contribute to earlier menopause, which is in keeping with previously reported data (9, 15, 22). Low socioeconomic status (SES) has been associated with an elevated risk of unhealthy behaviors (such as early initiation of smoking, high-energy food, and low physical activity), while high socioeconomic status was associated with healthy diet (such as suitable consumption of fruits, vegetables, and dairy products, regular breakfast) and active lifestyle (such as appropriate physical exercise) (30). As a result, living and eating habits are related with the ANM. In keeping with others’ reports (10, 31), we found smoking habit to be associated with earlier ANM and EM. The components of smoke may cause demise of oocytes by reactive oxygen species (ROS) and activating pro-apoptotic proteins [Bcl-2 and Bcl-2-associated X protein (BAX) proteins] (32, 33). Interestingly, we found that the habit of eating spicy foods could delay ANM by approximately 3.6 months, and the reason might be the antioxidant, anti-inflammatory, and immunomodulatory effects of chili pepper (34, 35). Furthermore, women who had experienced severe food shortage are more likely to experience EM, which was in keeping with previous reports (36, 37).

In this study, we found that older at survey was associated with later ANM, less POF and EM, and more LM. Other studies also reported that the long-term trend of ANM may be non-linear (38). In China, the policy of family planning, educational level, economic status, and food supply have differed between different generations (39)—for instance, the great famine during 1959–1961 (40). Since the reform in 1978, economy has progressed remarkably in China (41), which may have induced the connection between ANM and the age at survey.

After adjusting for the abovementioned confounders, the ANM of Han women was 0.74 year earlier than that of Tibetan women. The discrepancy may be attributed to the genetic background of the two ethnic groups. A number of genes have been associated with the ANM, with the range of effect ranging from 3.5 to 74 weeks (42). Ethnic heterogeneity of genes, such as *FRMD5* and *GPRC5B*, has been associated with the ANM among Asian and African women but not in Hispanic and Latino (Fernández-Rhodes et al., 2018). Another study has revealed differences in allelic frequencies of eight genes associated with the ANM, including 10q24.1/*CCNJ*, 3q21.3/*H1FX*, 4p11/*ZAR1*, 8p21.2/*GNRH1*, 18q21.33/*ZCCHC2*, 8q24.11/*RAD21*, 4q23/*EIF4E*, and 14q24.2/*DCAF4* between Japanese and European populations (43).

It should be acknowledged that many studies investigating ANM determinants have included BMI as a potential confounding factor in their regression models (3, 12). However, given the time-dependent variable of BMI which may fluctuate substantially over decades, to use postmenopausal BMI measurements (collected years or even decades after menopause) as an approximation for premenopausal BMI could introduce a significant measurement error (10). Furthermore, many studies examining the association between ANM and BMI have reported no significant correlation between them (3). For these methodological and evidence-based considerations, we have prudently chosen not to include BMI as a covariate in our analysis.

Several limitations of this study should be acknowledged. First, the retrospective assessment of ANM may be subject to recall bias, particularly among older participants. Although our sensitivity analysis excluding women aged >75 years yielded consistent results, residual misclassification cannot be completely ruled out. Second, while we adjusted for numerous covariates, unmeasured confounding factors such as genetic predisposition and detailed hormonal profiles may influence the observed ethnic differences. Third, due to the cross-sectional nature of design, causal relationships between identified factors and ANM cannot be established. Prospective studies with accurate ANM and more information on confounders should be carried out to uncover the relationship between the ANM and ethnicity.

## Data Availability

The raw data supporting the conclusions of this article will be made available by the authors, without undue reservation.
