# Supplementary material for: The age at natural menopause of Chinese Han and Tibetan women
Source: Front Endocrinol (Lausanne). 2025 Aug 1;16:1584267. doi: 10.3389/fendo.2025.1584267 (PMC12355183; doi:10.3389/fendo.2025.1584267)
Supplement: Supplementary file 1 [file Table1.docx]

**Supplementary Table S1 Exposure variables and operational definitions**

| Exposure variable | Operational definition | Variable category/presentation of data |
| --- | --- | --- |
| Ethnicity | Self-reporting ethnicity | Categories: Han, Tibetan |
| Age at survey, (year) | Age at the time of survey | Continuous term |
| Marriage status | Current marital status | Categories: Never married, Ever married (include married, widowed, separated and divorced) |
| Educational level | Highest educational level | Categories: Middle school and below, High school and above |
| Annual household income | Family’s total income before tax (RMB) | Categories: < 20,000, 20,000 – 99,999, 100,000-199,999, ≥ 200,000 |
| Smoking | Smoking habit | Categories: No smoking, Ever smoking (including smoking and quited smoking) |
| Passive smoking | “Have you passively inhaled smoke from cigarettes or other tobacco products in your home, workplace, or entertainment in the past?” | Categories: No, Yes |
| Alcohol intake | Drinking alcohol habit | Categories: Never drinking, Ever drinking (including occasional and regular drink alcohol) |
| Tea drinking | “Do you have the habit of drinking tea?” | Categories: No, Yes |
| Soft drinking | “Do you have the habit of drinking soft?” | Categories: No, Yes |
| Spicy foods | “Do you have the habit of eating spicy foods?” | Categories: No, Yes |
| Numb foods | “Do you have the habit of eating numb foods?” | Categories: No, Yes |
| Labor history | “Have you been engaged in farming or animal husbandry in the past year?” | Categories: No, Yes |
| Pesticide exposure history | “Have you regularly used or come into contact with pesticides in your life or work previously?” | Categories: No, Yes |
| Coal smoke exposure history | “Have you ever had smoke pollution from burning coal in your home, especially in winter?” | Categories: No, Yes |
| Severe food shortage history | “Have you ever experienced a severe food shortage in your life?” | Categories: No, Yes |
| Age at menarche, (year) | Age of first menstruation (menarche) | Continuous term |
| No. of pregnancy | Number of pregnancy | Continuous term |
| No. of parity | Number of live births | Continuous term |
| Age at first birth, (year) | Age at birth of first child | Continuous term |
| Duration of breastfeeding per child, (month) | Average time to breastfeed each child | Continuous term |
| Utilization of IUD | “Have you ever used an IUD?” | Categories: Never, Ever |
| Utilization of OC | “Have you ever taken oral contraceptive?” | Categories: Never, Ever |

Abbreviations: No., number; IUD, intrauterine device; OC, oral contraceptive.

**Supplementary Table S2 Multivariate Linear regression analysis of ANM (Age at survey ≤ 75 years old** ^a^**)**

| Variables | Multivariate analysis ^b^ | |
| --- | --- | --- |
|  | β (95%CI) | *P*-value |
| Ethnicity (Ref: Tibetan) | -0.67 (-1.17, -0.16) | 0.009 |
| Age at survey | 0.15 (0.13, 0.16) | < 0.001 |
| Marriage status (Ref: Never married) | 2.80 (0.99, 4.61) | 0.002 |
| Education level (Ref: Middle school and below) | 0.60 (0.27, 0.93) | < 0.001 |
| Annual household income (Ref: < 20000) |  |  |
| 20000 ~ 99999 | 0.20 (-0.04, 0.43) | 0.110 |
| 100000 ~ 199999 | 0.21 (-0.23, 0.66) | 0.350 |
| ≥ 200000 | 1.04 (0.09, 1.99) | 0.032 |
| Smoking (Ref: No smoking) | -0.37 (-1.18, 0.44) | 0.372 |
| Passive smoking (Ref: No) | 0.03 (-0.20, 0.25) | 0.830 |
| Alcohol intake (Ref: Never drinking) | -0.26 (-0.52,-0.00) | 0.047 |
| Tea drinking (Ref: No) | 0.27 (0.02, 0.51) | 0.033 |
| Soft drinking (Ref: No) | -0.43 (-0.90, 0.05) | 0.077 |
| Spicy foods (Ref: No) | 0.31 (0.05, 0.57) | 0.019 |
| Numb foods (Ref: No) | -0.03 (-0.30, 0.25) | 0.836 |
| Labor history (Ref: No) | 0.16 (-0.10, 0.41) | 0.223 |
| Pesticide exposure history (Ref: No) | 0.11 (-0.14, 0.37) | 0.381 |
| Coal smoke exposure history (Ref: No) | 0.02 (-0.21, 0.26) | 0.844 |
| Severe food shortage history (Ref: No) | -0.40 (-0.65, -0.15) | 0.002 |
| Age at menarche (year) | 0.04 (-0.01, 0.09) | 0.128 |
| No. of pregnancy | 0.10 (0.02, 0.17) | 0.013 |
| No. of parity | -0.31 (-0.45, -0.18) | < 0.001 |
| Age at first birth (year) | -0.01 (-0.05, 0.03) | 0.760 |
| Duration of breastfeeding per child (month) | 0.01 (-0.01, 0.02) | 0.606 |
| Utilization of IUD (Ref: Never) | 0.52 (0.28, 0.77) | < 0.001 |
| Utilization of OC (Ref: Never) | -0.39 (-0.73, -0.04) | 0.028 |

Abbreviations: CI, confidence interval; No., number; IUD, intrauterine device; OC, oral contraceptive.

^a^ postmenopausal women whose age at survey ≤ 75 years were 6353.

^b^ Adjusted for ethnicity, age at survey, marriage status, education level, annual income, smoking, passive smoking, alcohol intake, tea drinking, soft drinking, spicy foods, numb foods, labor history, pesticide exposure history, coal smoke exposure history, severe food shortages history, age at menarche, number of pregnancy, number of parity, age at first birth, duration of breastfeeding per child, utilization of IUD and utilization of OC, except for the same variables.

**Supplementary Table S3 Multinomial logistic regression for non-normal menopausal type (Age at survey ≤ 75** ^a^**)**

| Variable | POF ^b^ (n = 204) | | EM ^b^ (n = 715) | | LM ^b^ (n = 329) | |
| --- | --- | --- | --- | --- | --- | --- |
|  | *OR* (95% CI) | *P*-value | *OR* (95% CI) | *P*-value | OR (95% CI) | *P*-value |
| Ethnicity (Ref: Tibetan) | 1.14 (0.58, 2.23) | 0.703 | 1.32 (0.90, 1.94) | 0.151 | 0.53 (0.32, 0.88) | 0.013 |
| Age at survey | 0.91 (0.89, 0.93) | < 0.001 | 0.95 (0.94, 0.96) | < 0.001 | 1.10 (1.08, 1.12) | < 0.001 |
| Marriage status (Ref: Never married) | 0.35 (0.07, 1.70) | 0.192 | 0.32 (0.12, 0.90) | 0.030 | 1.15 (0.14, 9.30) | 0.893 |
| Education level (Ref: Middle school and below) | 0.86 (0.54, 1.37) | 0.520 | 0.69 (0.52, 0.90) | 0.007 | 1.15 (0.80, 1.65) | 0.452 |
| Annual household income (Ref: < 20000 RMB) |  |  |  |  |  |  |
| 20000 ~ 99999 | 0.77 (0.56, 1.07) | 0.118 | 0.96 (0.80, 1.15) | 0.678 | 0.92 (0.71, 1.20) | 0.534 |
| 100000 ~ 199999 | 0.79 (0.42, 1.50) | 0.469 | 0.99 (0.69, 1.41) | 0.948 | 1.25 (0.78, 1.98) | 0.354 |
| ≥ 200000 | 0.34 (0.05, 2.57) | 0.299 | 0.25 (0.06, 1.02) | 0.054 | 1.81 (0.81, 4.03) | 0.147 |
| Smoking (Ref: No smoking) | 1.11 (0.34, 3.62) | 0.864 | 1.43 (0.81, 2.54) | 0.214 | 0.95 (0.38, 2.42) | 0.923 |
| Passive smoking (Ref: No) | 0.95 (0.69, 1.32) | 0.768 | 1.12 (0.94, 1.34) | 0.201 | 0.84 (0.65, 1.09) | 0.202 |
| Alcohol intake (Ref: Never drinking) | 0.77 (0.52, 1.14) | 0.187 | 1.18 (0.96, 1.44) | 0.108 | 1.03 (0.77, 1.38) | 0.841 |
| Tea drinking (Ref: No) | 0.89 (0.63, 1.26) | 0.524 | 0.86 (0.71, 1.04) | 0.129 | 1.10 (0.85, 1.44) | 0.463 |
| Soft drinking (Ref: No) | 1.16 (0.66, 2.04) | 0.609 | 0.96(0.67, 1.35) | 0.797 | 0.62 (0.35, 1.10) | 0.104 |
| Spicy foods (Ref: No) | 0.85 (0.59, 1.22) | 0.367 | 0.81 (0.67, 0.99) | 0.039 | 1.00 (0.76, 1.32) | 0.997 |
| Numb foods (Ref: No) | 1.20 (0.82, 1.77) | 0.341 | 0.95 (0.77, 1.17) | 0.643 | 0.90 (0.67, 1.21) | 0.482 |
| Labor history (Ref: No) | 0.84 (0.60, 1.18) | 0.310 | 0.98 (0.81, 1.19) | 0.877 | 0.82 (0.62, 1.09) | 0.167 |
| Pesticide exposure history (Ref: No) | 0.76 (0.52, 1.10) | 0.144 | 1.01 (0.83, 1.23) | 0.939 | 0.91 (0.68, 1.20) | 0.490 |
| Coal smoke exposure history (Ref: No) | 0.90 (0.64, 1.26) | 0.532 | 0.94 (0.78, 1.13) | 0.494 | 1.06 (0.82, 1.36) | 0.678 |
| Severe food shortage history (Ref: No) | 1.30 (0.90, 1.89) | 0.164 | 1.27 (1.05, 1.55) | 0.016 | 0.87 (0.66, 1.13) | 0.286 |
| Age at menarche (year) | 1.01 (0.94, 1.09) | 0.706 | 1.00 (0.96, 1.04) | 0.865 | 1.02 (0.97, 1.08) | 0.440 |
| Number of pregnancy | 1.03 (0.92, 1.14) | 0.625 | 0.97 (0.91, 1.03) | 0.298 | 1.04 (0.97, 1.12) | 0.289 |
| Number of parity | 1.07 (0.90, 1.28) | 0.426 | 1.21 (1.09, 1.33) | < 0.001 | 0.89 (0.78, 1.02) | 0.100 |
| Age at first birth (year) | 1.01 (0.95, 1.07) | 0.760 | 1.00 (0.97, 1.04) | 0.797 | 1.03 (0.99, 1.08) | 0.112 |
| Duration of breastfeeding per child (month) | 1.00 (0.97, 1.03) | 0.923 | 1.00 (0.99, 1.02) | 0.940 | 1.00 (0.98, 1.02) | 0.889 |
| Utilization of IUD (Ref: Never) | 0.98 (0.69, 1.38) | 0.900 | 0.74 (0.61, 0.89) | 0.001 | 1.01 (0.78, 1.31) | 0.932 |
| Utilization of OC (Ref: Never) | 1.31 (0.83, 2.05) | 0.241 | 1.44 (1.12, 1.85) | 0.004 | 1.23 (0.85, 1.78) | 0.266 |

Abbreviations: CI, confidence interval; POF, premature ovarian failure; EM, early menopause; LM, late menopause. OR, odds ratio; No., number; IUD, intrauterine device; OC, oral contraceptive.

^a^ postmenopausal women whose age at survey ≤ 75 years were 6353.

^b^ Adjusted for ethnicity, age at survey, marriage status, education level, annual income, smoking, passive smoking, alcohol intake, tea drinking, soft drinking, spicy foods, numb foods, labor history, pesticide exposure history, coal smoke exposure history, severe food shortage history, age at menarche, number of pregnancy, number of parity, age at first birth, duration of breastfeeding per child, utilization of IUD and utilization of OC, except for the same variable.
